# Multitemporal Mapping of Post-Fire Land Cover Using Multiplatform PRISMA Hyperspectral and Sentinel-UAV Multispectral Data: Insights from Case Studies in Portugal and Italy

**DOI:** 10.3390/s21123982

**Published:** 2021-06-09

**Authors:** Giacomo Lazzeri, William Frodella, Guglielmo Rossi, Sandro Moretti

**Affiliations:** 1 Department of Earth Sciences, University of Firenze, Via La Pira 4, 50121 Firenze, Italy; William.frodella@unifi.it (W.F.); sandro.moretti@unifi.it (S.M.); 2 Centre of Civil Protection, University of Florence, Largo Fermi 1, 50125 Firenze, Italy; Guglielmo.rossi@unifi.it

**Keywords:** remote sensing, hyperspectral, multispectral, vegetation recovery, burn severity, soil charring, drone, scene classification, RBR

## Abstract

Wildfires have affected global forests and the Mediterranean area with increasing recurrency and intensity in the last years, with climate change resulting in reduced precipitations and higher temperatures. To assess the impact of wildfires on the environment, burned area mapping has become progressively more relevant. Initially carried out via field sketches, the advent of satellite remote sensing opened new possibilities, reducing the cost uncertainty and safety of the previous techniques. In the present study an experimental methodology was adopted to test the potential of advanced remote sensing techniques such as multispectral Sentinel-2, PRISMA hyperspectral satellite, and UAV (unmanned aerial vehicle) remotely-sensed data for the multitemporal mapping of burned areas by soil–vegetation recovery analysis in two test sites in Portugal and Italy. In case study one, innovative multiplatform data classification was performed with the correlation between Sentinel-2 RBR (relativized burn ratio) fire severity classes and the scene hyperspectral signature, performed with a pixel-by-pixel comparison leading to a converging classification. In the adopted methodology, RBR burned area analysis and vegetation recovery was tested for accordance with biophysical vegetation parameters (LAI, fCover, and fAPAR). In case study two, a UAV-sensed NDVI index was adopted for high-resolution mapping data collection. At a large scale, the Sentinel-2 RBR index proved to be efficient for burned area analysis, from both fire severity and vegetation recovery phenomena perspectives. Despite the elapsed time between the event and the acquisition, PRISMA hyperspectral converging classification based on Sentinel-2 was able to detect and discriminate different spectral signatures corresponding to different fire severity classes. At a slope scale, the UAV platform proved to be an effective tool for mapping and characterizing the burned area, giving clear advantage with respect to filed GPS mapping. Results highlighted that UAV platforms, if equipped with a hyperspectral sensor and used in a synergistic approach with PRISMA, would create a useful tool for satellite acquired data scene classification, allowing for the acquisition of a ground truth.

## 1. Introduction

Amongst natural hazards, wildfire has had natural functions in the ecosystem’s regulation since the Quaternary [1] and was part of the evolution of man since the Paleolithic, whereas currently it is damaging natural resources and creating widespread socio-economic damage [2]. In the Mediterranean basin this is due to the abandonment of the rural areas and a decrease in the land managing practices starting from 1960 [3]. The cessation of these practices has resulted in an afforestation with flammable species and an increase of vegetation fuel loads over time [4]. Indeed, fuel types affect fire behavior, as not all fuel burns in the same way [5]. This fact and the prevalence of flammable species (e.g., pine, eucalyptus) combined with changes in rainfall regimes and temperatures arising from climate change has contributed to the increase of wildfire intensity [3,6]. On a landscape scale, apart from fuel load management and the reduction of human-caused ignitions via awareness rising, nothing can be done to prevent wildfire occurrence, since it is impossible to modify its influencing parameters such as terrain shape, wind, and drought.

### 1.1. Limitatons of Field Method

**Figure 1:**
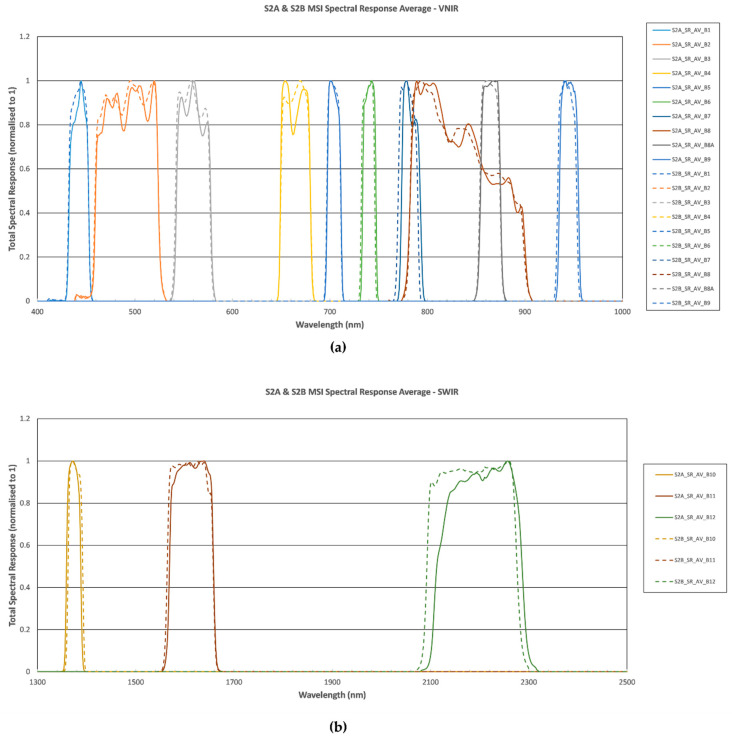
Sentinel-2 bands and their spectral sampling intervals for VNIR (400–1000 nm) and SWIR (1000–2500 nm) [21].

Before the advent of remote sensing, traditional methods for burned area mapping consisted of delimiting the perimeter of the area of interest by using GPS devices. This protocol showed its limitation in scenarios of harsh inaccessible morphology, still active wildfires, or uneven burning patches. Furthermore, the delimitation of the area is time-consuming and very expensive as it needs trained personnel [7]. Additionally, field methods provide limited spatial and temporal representation of post-fire ecological effects [8]. In the field of earth observation, fire causes spectral changes of the scenario with the charring of vegetation, thermal changes of land surface, and the general consumption of vegetation [9]. Likewise, fire reduces aboveground and belowground moisture [10,11]. The reduction of chlorophyll absorption determines an increased reflectance in the visible electromagnetic region, coupled with leaf tissue damage, resulting in decreased reflectance in the near infrared (NIR) region [12,13]. Fire is a strong agent able to alter the observed scenario in a wide range of severity, and therefore modifying the features spectral signature. Therefore, to exploit these spectral variations, complex and multitemporal indices have been developed: NDVI (normalized difference vegetation index), dNDVI (delta NDVI), NBR (normalized burn ratio), dNBR (delta NBR), RBR (relativized burn ratio), and BIAS2 (burned area index for Sentinel-2), coupled with vegetation biophysical indices: fCover (fraction of green vegetation cover), LAI (leaf area index), and fAPAR (fraction of absorbed photosynthetically active radiation). Compared to the pre-satellite era, burned areas were mapped via field sketching or biome-aggregated best-guess values of the average annual area burned [14,15]. Burned area information has gained relevance over the years due to the stipulation of international contracts related to fire emissions, such as the Kyoto protocol and the UN Sustainable Development Goals (SDGs) [16,17]. The development of satellite-based burned area index computation has allowed for a more precise world burned area assessment. The priority in assessing the area affected by wildfires is linked to the necessity of adopting practices for the reduction of the ecosystem impact, such as soil erosion and biodiversity reduction, and to assess the emission of greenhouse gasses (as CO^2^) coming from the biomass combustion. In the Italian context, attention is focused on the nature of fire as it is often used as a mean for uncontrolled edification [18]. At the European level, the most important group operating in the wildfire monitoring and prevention is EFFIS (European Forest Fire Information System). Developed by the European Joint Research Centre, this system is part of the Copernicus complex and multi-thematic earth observation program. Copernicus is a revolutionary satellite constellation dedicated to earth observation and monitoring [19], implemented to tackle the dispersion of founding and knowledge by fragmented European earth observation activities, providing the union members a communitarian portal for Earth observation free and opensource products. Within the thirty-plus satellites making up the Copernicus constellation, the Sentinel family is constituted by six new satellites equipped with different sensor platforms for earth observation, both sea and land, granting a small revisit period over Europe [20]. More specifically, in the field of land cover analysis, Sentinel-2 is a performing platform as it embarks a multispectral sensing instrument (MSI), acquiring thirteen bands in the optic to SWIR range, with particular focus on the red-edge band, one of the best chlorophyll content descriptors useful for vegetation analysis, Figure 1 [21]. Due to these characteristics, Sentinel-2 has proved to be a performing platform for burned area mapping, both individually [22,23,24] and paired with other sensors [25,26,27].

Differently from Sentinel, PRISMA satellite (hyperspectral precursor of the application mission) is a recently introduced earth observation system launched by ASI (Italian Space Agency), equipped with an innovative electro-optic hyperspectral sensor capable of chemical scene characterization [28]. The sensor high spectral resolution allows for environmental analysis, ranging from geological mapping, vegetation analysis, and pollutant diffusion [29,30,31].

### 1.2. Sensing Platforms for the Mapping of Burned Areas

First approaches to burned area mapping were recorded by Sharpe in 1920 for province-wide forest surveys [32] and consisted of aerial sketching of the fire perimeter. Further developments consisted of the fire perimeter GPS acquisition by field tailing or by helicopter hoovering [33]. Nevertheless, this methodology lacked in accuracy in topographically dissected areas and exposed the field personnel to risks related to recently burned areas. These criticalities were overcome with the deployment of Landsat 1 in 1972, allowing for the accurate definition of recent forest fire boundaries and spectral class discrimination [14,34]. The academic research about post-fire area detection through multispectral satellite remote sensing is well developed and exhaustive [22,35,36,37,38,39,40,41,42].

Currently, burned area analysis and mapping is usually performed through satellite remotely sensed data, providing efficient and reliable results for data collected over clear portions of sky. This can be done for fires with an extent greater than the sensor spatial resolution and for areas with a consistent aspect and morphology [14]. In recent years, additional development for burned area mapping came with the implementation of UAV methodologies, i.e., unmanned aerial vehicles with remote piloting. When compared to satellite sensing, this new platform allows for greater spatial and temporal resolution, increasing the mapping accuracy for uneven burning paths and fire perimeter areas [7,43,44]. Depending on the purpose of the satellite, different platforms will have different sensors able to take advantage of the objective spectral response.

#### 1.2.1. Sentinel-2 Platform

The launch of Sentinel-2 satellites equipped with multi-spectral instrument (MSI) sensors characterized by specific spectral bands configuration, combined with its short revisit time, has opened possibilities for the development and application of new indices for fire burnt severity assessment [45]. The sensor is equipped with the best radiance-based descriptors of chlorophyll content [46] and its wide spectral range allows for soil characterization in the VIS-NIR-SWIR range, providing sufficient accuracy to investigate soil type and cover [47]. Listed in Table 1 are Sentinel-2 spectral bands and their purpose. Different bands have different spatial resolutions because of the detail needed to analyze the objective and its interaction with the electromagnetic radiation.

#### 1.2.2. Prisma Platform

Prisma is a cutting-edge earth observation system developed by ASI launched in 2019 and operative since May 2020 [48]. It is equipped with a medium resolution panchromatic camera (5 m) and a two spectrometer (VNIR and SWIR) hyperspectral sensor, capable of characterizing the scene both optically and chemo-physically [28]. The double spectrometer configuration allows for a high spectral resolution over a wide sensing range (400–2505 nm), which is also due to a 10-nm spectral sensing interval (SSI). Such configuration allows a spectrometer ground spatial resolution of 30 × 45 m to be obtained, Table 2 [49].

#### 1.2.3. UAV Platform

Drones or UAVs (unmanned aerial vehicle) are currently used in a wide spectrum of applications, ranging from aerial photography to military purposes. The ability to equip these aircrafts with scientific instruments opens a totally new approach to spatial analysis [44]. Applications range from forest characterization for tree species and wildlife monitoring [50,51], estimation of post-fire vegetation recovery [7,43,44], and landslide photogrammetric analysis [52,53].

Low altitude flights provide ultra-high spatial resolution and allow single feature analysis of the considered phenomenon. In addition, the cost of data collection is much lower and safer compared to ground data collection. The unmanned aircraft used in this work falls in the “light” UAV class (<25 kg take-off weight) and allows a hoovering time of 30 min. Most of the commercial drones have relatively low carrying capacity and stability. Further UAV specifications are provided in [54]. In this study, the analysis of the area of interest was performed through a customized Canon Powershot S100 digital camera with a filter for the collection of NIR radiation [55].

### 1.3. The Advantages and Development of Remote Sensing Methods

The application of satellite-sensed data for earth observation purposes might hold biases and inaccuracies arising from the distance and atmosphere between the platform and the objective. For this reason, due to its modularity and quick deployment, currently unmanned aerials provide a cost-effective solution for the validation of satellite data at a detailed scale. In the context of land cover and fire severity analysis, UAVs equipped with specific sensors can provide a useful mean to gain insights in areas were satellite mapping proved to be less accurate, such as the burned area perimeters and uneven burned patches [56,57].

The present study focuses on multitemporal burned area perimeter mapping by analyzing the soil–vegetation recovery phenomena for fire affected areas via Sentinel-2 and PRISMA remotely sensed data. To assess the potentialities of the spaceborne data, the obtained results were compared with UAV acquired data to determine the benefit resulting in the higher resolution of the platform and provide validation for the satellite-sensed indices. Two study sites, Portugal and Italy, were chosen because of their different aspects and severity. The very different aerial extent of the two events allowed for site-based technique development.

## 2. Burned Area Mapping and Vegetation Indices

### 2.1. Burned Area Indices

Fire changes the landscape and disrupts the existing vegetation pattern, therefore, in order to assess the extent of this impact, a valuable solution is provided by the chlorophyll pigment absorption analysis [58]. This pigment electromagnetic spectrum absorption peaks for the blue and red corresponding wavelengths, while being highly reflective in the green portion [59]. Furthermore, the spongy structure of the mesophyll tissue and cavities within the leaf determines high NIR reflectance [60]. As soil’s main absorption is in the blue region of the spectrum, this facilitates its distinction with respect to vegetation, allowing for the combination of these different bands in newly computed indices [60]. To assess postfire scar extent and vegetation recovery of the fire affected area, a multitemporal approach can be adopted [22]. Multitemporal observations gather better results when compared to single temporal observations as they rely on the estimation of changing vegetation cover. This technique is based on the computation operated via specifically developed indices: e.g., the delta normalized burn ratio (dNBR) [59], the relativized burn ratio (RBR) [61], the relative differenced normalized burn ratio (RdNBR) [62], or the bun area index for Sentinel-2 (BAIS2) [22], and all can be profitably used for burned area severity assessment.

Although no agreement exists on which index performs best in the detection of fire-affected areas and for fire severity assessment, according to Parks, Dillon, and Miller [61], when averaged among different scenarios, RBR proved to have the highest classification accuracy in comparison to RdNBR and dNBR. Additionally, as with RdNBR, RBR has the capability to detect change in canopy cover over multitemporal images also for low vegetated areas. When compared to the other indices, the improved performance of RBR coupled with the detailed process implementation from ESA Research and User Support (RUS) [63] provides solid ground for the data processing and the index selection.

### 2.2. Vegetation Indices

Despite the capability of RBR to also record vegetation recovery after the event, to gather detailed information about biophysical parameters, specific index computation procedures were developed in the framework of Sentinel-2 data processing. These procedures were supported by the evidence in the potential of Sentinel-2 red-edge spectral domain devoted band (B8A) data for retrieving leaf area index (LAI) derived parameters [64].

Indices can be calculated with simple empirical regressions, which are efficient but imprecise, or by analyzing vegetation response using opensource toolboxes, e.g., SNAP [65], QGIS [66] equipped with a complex radiative transfer model inversions algorithm based on strong assumptions, and a turbid model conceptualization for vegetation [67]. Weiss and Baret [67] implemented the SNAP exclusive biophysical parameter retrieval toolbox as a collection of backpropagation artificial neural networks (ANN), trained using a globally representative set of simulations from a canopy radiative transfer model (RT), resulting in PROSAIL. This model allows for both the simulation of the top of canopy reflectance and the computation of secondary biophysical variables closely related to each other, as a fraction of vegetation cover (fCover or FVC), leaf area index (LAI), and fraction of absorbed photosynthetically active radiation (fAPAR).

Leaf area index (LAI), a dimensionless ratio of the green leaf area, is defined as half the developed area of photosynthetically active elements of the vegetation per unit horizontal ground area canopy [67]. Remotely sensed vegetation LAI will include the green contribution of the forest understory. Furthermore, it refers to green parts of the canopy with leaf chlorophyll content higher than 15 µg × cm^−2^ [67]. fAPAR is a direct measure of the proportion of incoming photosynthetically active radiation (400–700 nm) absorbed by the canopy. In the toolbox model it directly derives from the radiative transfer model in the canopy. Regarding fCover, this parameter is an indicator of plant growth during early periods before the soil is fully canopy-covered. It corresponds to the gap fraction of vegetation for the nadir direction, and it is often used to separate vegetation and soil in energy balance processes for evapotranspiration computation. Similarly to LAI, both fCover and fAPAR are only sensitive for chlorophyll contents higher than 15 µg × cm^−2^.

## 3. Materials and Methods

### 3.1. Case Studies

#### 3.1.1. Castanheira de Pêra Fire—Study Site 1

The Portuguese municipality of Castanheira de Pêra in the Leiria district (40°03′35.2′′ N 8°12′26.4′′ W), (Figure 2a–d) in June 2017 suffered a severe fire that burnt more than 53,000 hectares of land, killing sixty-six people and requiring eight days to be extinguished. Soil originated from the alteration of pre-Ordovician schist of the Hesperic Massif, classified as Cambisol using the international soil classification system [68]. The area is located 817 m a.s.l. and is characterized by a 40–50-year-old *Pinus pinaster* vegetation (field evaluated). The general aspect varies from W to SW, with an average slope of 15°. The climate is Atlantic with a Mediterranean influence, classified as Csb by the Köppen–Geiger system. The average annual temperature is 14.2 °C and the rainfall averages 1092 mm [69]. The field evaluation for soil burnt severity assessment was conducted following the method and classes by Vega et al. [70]. Given the extreme fire extension, to streamline the process we selected an area of interest where the average fire severity was mapped, resulting in a medium severity classification. Generally, all fire severity classes were represented within the fire perimeter.

#### 3.1.2. Vinchiana Fire—Study Site 2

**Figure 2:**
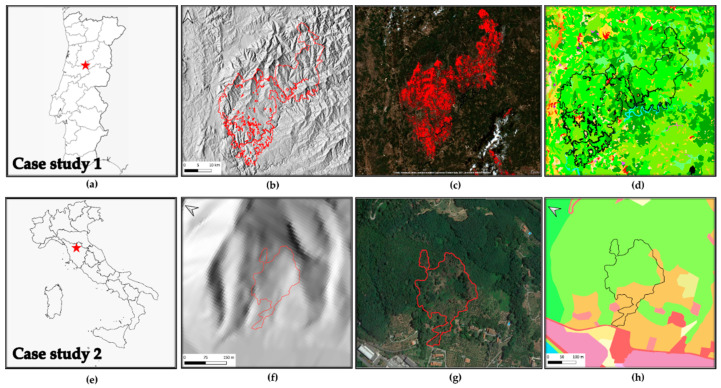
Case study 1: (**a**) location of the study site in Portugal; (**b**) digital elevation model of the study area 25 × 25 m [73], (**c**) Copernicus sentinel hub burned area [74], (**d**) 2012 Corine land cover [75], legend accessible at [76]. Case study 2: (**e**) location of the study site in Italy; (**f**) digital elevation model, (**g**) Google earth view of wildfire burned area, (**h**) Tuscan database for landcover, legend accessible at [77].

The Vinchiana fire occurred in Italy, in the Tuscany region (Figure 2e–h), and was reported on the 8th of August 2018 and burned 3.6 hectares. The fire started in the cultivated area and spread to the woods with a canopy burning path [71]. The propagation of fire was stopped upslope on the crest line, while its lateral extent was limited due to the intervention of fire fighters and the steep lateral valley lines [71]. Fortunately, the fire did not cause any life loss and spared the local houses.

The geology of the area is characterized by the Macigno Formation, a siliciclastic sandstone classified as graywacke, draped on top by Holocene eluvial and colluvial deposits farmed with olive tree cultivations and deciduous forests. The hill portion affected by fire has a general aspect-oriented S to W, with an average slope of 16°. The average annual temperature is 13.9 °C and the rainfall averages 1089 mm classified as Csa by the Köppen–Geiger system [72].

## 4. Methodological Approach

### 4.1. Study Site 1

**Figure 3:**
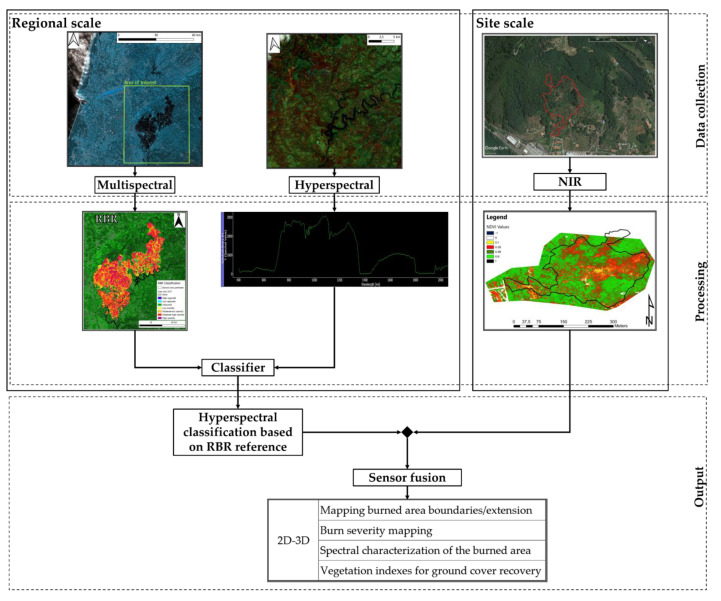
Flowchart of the methodology adopted for Sentinel-2, PRISMA, and UAV data analysis.

In order to account for the data variety and specificity, the image processing was performed via multiple software. Software as SNAP by ESA provide a rich suite of available tools for Sentinel-specific data processing. Nevertheless, to properly represent the data and visualize them, the best solution found was a secondary processing in a geoinformatics software, QGIS [66]. The calibration of the products parameters allowed the best representation possible via a dedicated color palette, Figure 3. Similarly, PRISMA data required the adoption of specifically developed hyperspectral analysis software, EnMap Toolbox [78], a graphic interface operating in Python code based on QGIS platform. The toolbox operational nature is centered on hyperspectral data analysis, in detail deriving from the German hyperspectral sensor for Environmental Mapping and Analysis Program (EnMAP), Figure 3.

#### 4.1.1. Relativized Burn Ratio (RBR) Computation

Sentinel-2 data was used to assess the wildfire burned area severity and extent with a particular focus on vegetation. A multitemporal approach has been adopted to compare the evolution of vegetation recovery and resprouting, and to assess the RBR index temporal variation. Multitemporal observations gather better results compared to singular observations as they rely on a non-affected data as a baseline to further asses change. The computation of the index has been handled on SNAP software. Analysis was performed on twelve Sentinel-2 L2A images ranging from 6 July 2017 (pre-fire acquisition) to 3 August 2019 (Table 3), with a time step of three months. Atmospheric correction was not necessary as Level 2A data were already calibrated for TOA and BOA reflectance. Nevertheless, to obtain a more robust result, the data were filtered to remove clouds and aerosol, elements that influence the index sensitivity and reliability. To avoid any excessive data corruption, the Sentinel images were selected according to a cloud cover threshold, and only data with under 5% cloud cover were used. The same procedure would have been implemented for PRISMA data; however, the possibility for the users to schedule acquisitions on specific areas does not allow a multitemporal analysis without a previously planned area of interest. Therefore, being the only available image for the area being acquired almost two years after the analyzed fire occurrence, and being the sensor extremely new in the spatial sensing constellation, it was decided to assess its capability to detect burned area spectral signature evidence despite the elapsed time. This objective was reached via the application of EnMap Toolbox [78].

The burned area and fire severity computation procedure was adapted from the methodology proposed by [63] and followed these steps:

1. Creation and subtraction to the acquisition of a layer constituted by the pixels classified as clouds and cirrus by Sentinel-2 preprocessed metadata;
2. Resampling of the bands to homogenize the spatial resolution to 10 m;
3. Computation of *NBR* index for each image as it is needed for RBR index computation:
  (1) $$NBR=\frac{NIR-SWIR}{NIR+SWIR}$$
4. Computation of the normalized difference water index (*NDWI*) [79] to reduce the noise arising from water bodies light scattering and subtraction from the images. The computation of this index was performed in the optic of improving the data quality or further *RBR* index computation:
  (2) $$NDWI=\frac{Green-NIR}{Green+NIR}$$
5. Computation of the *dNBR* index via subtraction among two adjacent acquisitions starting with the pre-fire image (couplets, Table 3):
  (3) $$dNBR=NBR_{pre-fire}-NBR_{post-fire}$$
6. Computation of the relativized burn ration (*RBR*) for each couplet:
  (4) $$RBR=\left(\frac{NBR_{pre-fire}-NBR_{post-fire}}{NBR_{pre-fire}+1001}\right)$$

The products resulting from this operation have been exported in the GeoTiff file for further visualization and analysis in QGIS. The products’ value can vary from case to case, making field validation a useful tool to assess the index accuracy. However, the United States Geological Survey (USGS) proposed a standardized classification table to interpret burn severity (Table 4). This value parametrization has been applied to the computed RBR images for visual representation.

#### 4.1.2. Biophysical Parameters Computation

**Figure 4:**
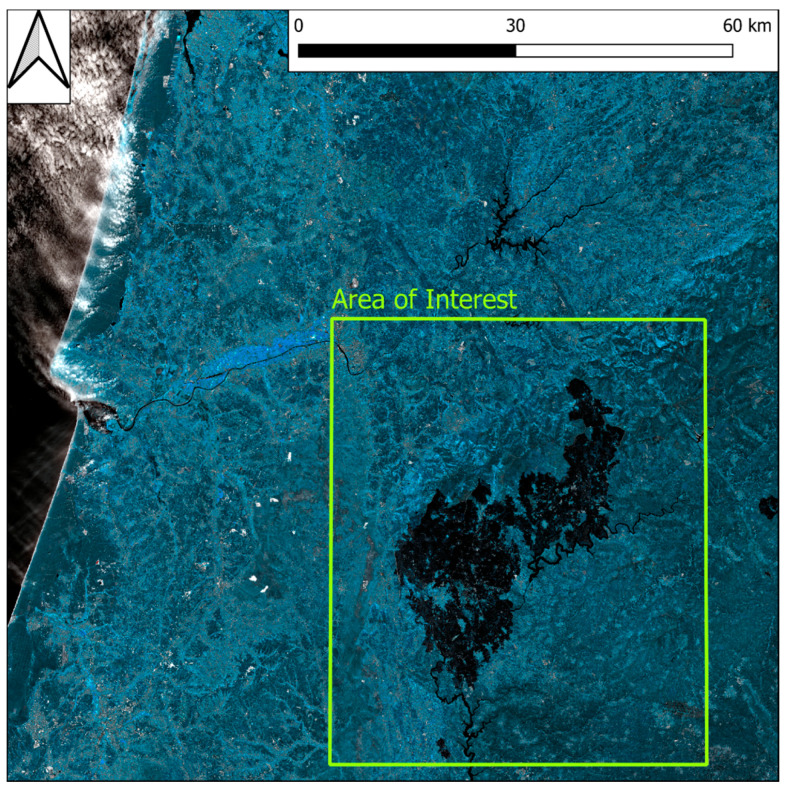
Area of interest (AoI) for biophysical parameter computation compared to Sentinel-2 granule dimension. In transparency is possible to observe the fire affected area. Image acquired on 4 July 2017.

SNAP provides a built in Sentinel-2 biophysical data analysis tool developed from Weiss and Baret [67]. The execution of it computes the leaf area index (LAI) and its subproducts as Cab (chlorophyll content in the leaf), Cw (leaf water content), fAPAR (fraction of absorbed photosynthetically active radiation), and fCover (vegetation cover fraction), used to separate vegetation and soil in energy balance, processes providing an excellent tool for green vegetation monitoring [67]. The operation has been executed for all the images in the dataset. The amount of data and band pre-processing performed for the new index computation turns in a burdensome computational effort. To partially overcome this issue, an area of interest around the fire-affected region, smaller than the Sentinel-2 acquisition, was designed, reducing the extent of the image (Figure 4).

The obtained outputs of fCover, fAPAR, and LAI values were singularly plotted per acquisition to observe the indices’ temporal variation. With this operation, it was possible to observe the biophysical index temporal variation before and after the wildfire, stressing the vegetation resprouting and health status, and taking as reference a non-fire-affected point and comparing it with the one acquired in a medium fire severity area.

#### 4.1.3. Hyperspectral Burned Scar Detection Assessment

Hyperspectral sensors proved to be efficient in burned area analysis, mapping the diffusion of ashes, coal and soil oxidation [81]. The PRISMA sensor used represents novelty and innovation due to its high spectral resolution. Nevertheless, in this study, the advantages this characteristic would have provide were almost nullified because of the time elapsed between the fire and the acquisition. Indeed, PRISMA was deployed on March 2019 while the fire occurred in June 2017. This delay allowed a significant scene change in comparison to the immediately post fire condition, creating a strong phase mixing within the pixel. The first PRISMA image over the AoI was acquired two years after the event and coincides with the last Sentinel-2 image. The AoI image was processed via EnMap Box, a QGIS plugin developed by the German Aereospatial agency in collaboration with Humboldt Universität of Berlin [78]. The tool is capable of opening PRISMA HDF5 files with the correlated ancillary files necessary for further processing. The PRISMA image was opened in the software and coregistered with Sentinel-2 burn severity RBR June–July 2017 product, used as a fire severity reference map.

**Figure 5:**
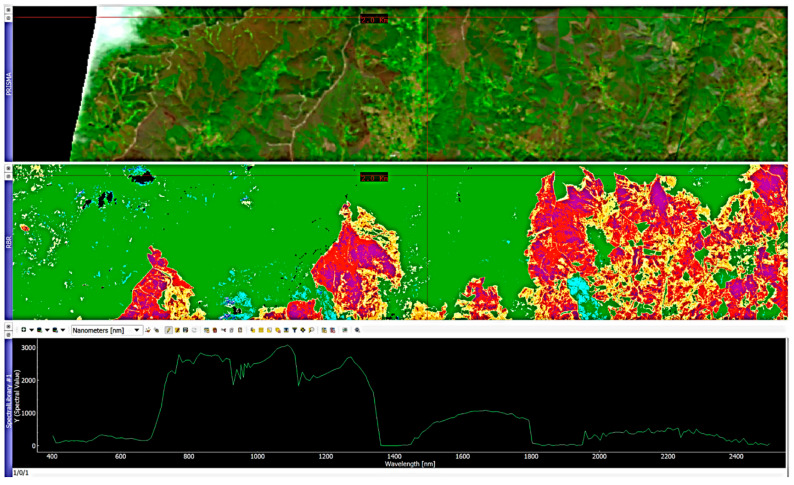
EnMap toolbox composite view, PRISMA, RBR, and spectral library.

This allowed a PRISMA feature spectral library to be created based on the acquisition of crosshair pointed ground pixel spectral signatures, classified with the reference of the 2017 Sentinel-2 burn severity map. This process allowed a classified spectral library to be created, associating a burn severity value of the area from the 2017 RBR map to a spectral signature of a 2019 PRISMA image (Figure 5). One hundred pixel-spectral signatures were acquired. The obtained classified spectral library was used as reference for the classification of the whole PRISMA scene via the EnMap toolbox, based on a random tree forest model. The sensor 237 spectral bands constitute a wide range of possible pixel spectral signatures, therefore requiring a remarkable computational power to perform the classification and definition of each pixel afference class.

### 4.2. Study Site 2

#### UAV NDVI Burned Area Mapping

The UAV surveys were performed on 13 November 2018 from 12:00 to 16:00, to simulate a quick response and reproductible procedure for data acquisition in wildfire-burned areas. The drone sampled the area at a height of 60 m above the hillside collecting two sets of data. The first flight was operated with the NIR sensor perpendicularly oriented to the horizon. During this sampling, the weather conditions were optimal due to a high sun angle, almost absent haze and low dew point. In the second data set collection, the sensor was tilted 30° to the horizon. The coordinates of the ground markers, necessary to increase the precision of the image overlapping, were acquired using a GPS field mapper. The base-rover method was used for the markers georeferencing.

The acquired data were processed via Agisoft PhotoScan Pro [82], creating a mosaic of the hovered area over which the NDVI_blue_ index (normalized difference vegetation index) was computed. The NDVI orthophoto was processed for the creation of a dense point cloud of the area to tridimensionally visualize the index.

## 5. Results

### 5.1. Study Site 1

#### 5.1.1. Relativized Burn Ratio (RBR) Computation

**Figure 6:**
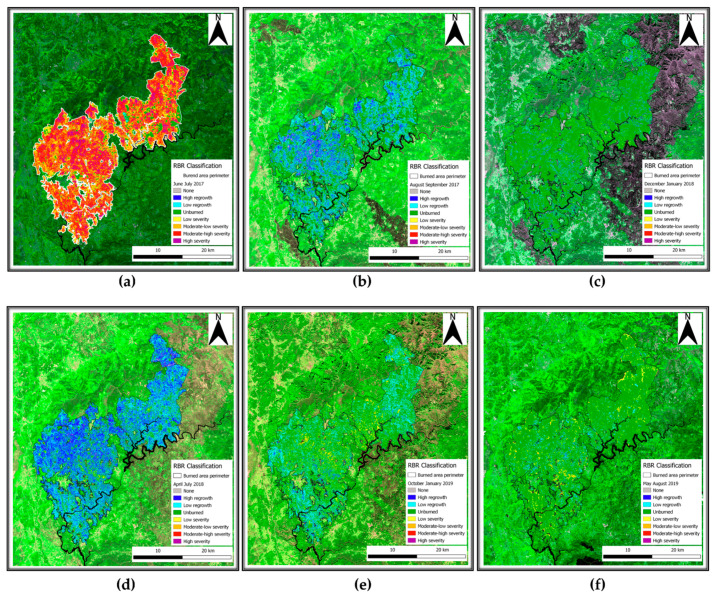
Sentinel-2 RBR computed index severity classes and area represented over the scene. (**a**) June–July 2017 couplet; (**b**) August–September 2017 couplet; (**c**) December–January 2018 couplet; (**d**) April–July 2018 couplet; (**e**) October–January 2019 couplet; (**f**) May–August 2019 couplet. 5-8A-5 color composite.

The RBR index for the June–July period showed an abundance of moderate–high and high severity areas with randomly scattered unburnt parcels within it. The index proved its accuracy highlighting the severity variation, from high to low and unburned, at the fire borders, reporting the effect of vegetation removal operated by fire (Figure 6a,b). Indeed, the destruction of the majority of vegetation leaves room for grass and bushes to thrive right after the event. This phenomenon is more evident for high-severity parcels but is not homogeneous across the burned area as it can be considered as a burst deriving from the removal of competitors. Additionally, we can exclude the observed regrowth to be an index contamination resulting from water vapor or scene illumination, since the phenomenon of resprouting does not carry on in the following December–January index computation (Figure 6c). Moreover, Figure 6c highlights the seasonal phenological dormant stage of vegetation. The presence of such extended areas classified as unburned patches can be addressed to the small or almost null variation in the elements as chlorophyll content and leaf structure, since the index is sensitive to these elements. The represented area also suffered further burning as testified by the dark scars highlighted via the index color composite. It is possible to appreciate the general and widespread vegetation resprouting, pointing out a general increase in ground cover both by forest understory and canopy area. Comparatively, observing the burn scar not included in the case study E to the analysis area (Figure 6c,d), we can observe how index color composites are a practical tool for expeditious optical analysis but are strongly influenced by the acquisition time in respect to the event. As the vegetation follows its phenological stages and the canopy cover seasonally variates, in winter (Figure 6e), we can appreciate how the index is capable of representing areas that still suffer low ground cover as inferred by parcels classified as low burn severity. In Figure 6f, the absence of evident change can be addressed by two main factors. The former can be considered the elapsed time since the fire. Indeed, as two years passed from the event, vegetation already re-established and reached a point for which its change is no longer detectable by RBR. The latter reason can be due to the effective absence of change in the time elapsed between May and August acquisitions.

#### 5.1.2. Biophysical Parameters Computation

**Figure 7:**
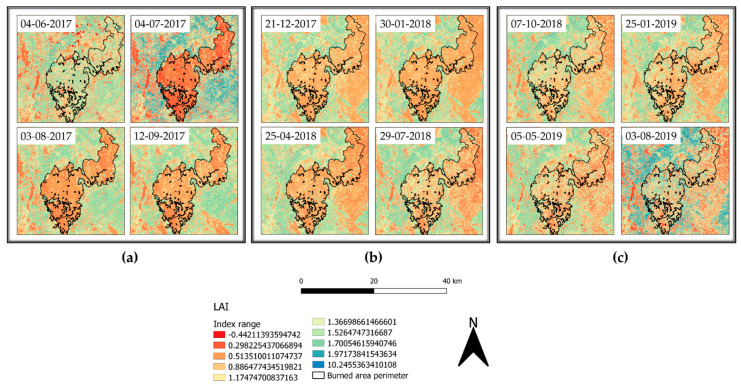
LAI index representation from 4 June 2017 to 3 August 2019.

**Figure 8:**
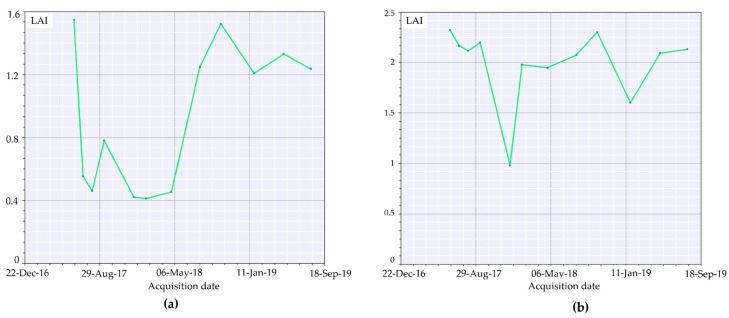
LAI index timeseries computed for: (**a**) the burned area (medium severity); (**b**) unburned area.

Image analysis conducted as reported in the materials and method section of this study highlighted the great efficiency for RBR to assess the soil burn severity and its variation over time. Indeed, the possibility to also observe the partial vegetation regrowth via the same index reduces the complexities deriving from the comparison of different indices. However, despite the information provided regarding vegetation health and resprouting, RBR nature and aim was not focused on vegetation health analysis. Therefore, biophysical indices were computed to fill the gap between fire burnt severity and vegetation recovery in multitemporal observations to assess the variation of vegetation health and ground cover. SNAP allowed for the computation of LAI, fCover, and fAPAR. For each index, the higher the value, the healthier and stronger the vegetation resprouting was evaluated. The LAI index, defined as the green leaf area per unit horizontal ground area, made it possible to observe through the acquisitions (Figure 7a–c) the progressive vegetation restoration highlighted by the index value variations. Despite the index being reported to range between one and eight, to favor the data visualization it was decided to represent the values within the 1% and 99% range. This decision was also supported by the presence of multiple fires surrounding the investigated area contributing for the index saturation, therefore reducing the information available on the area of interest (Figure 7a, 4 July 2017 acquisition). The timeseries graphic computed for LAI values for the burned area exhibited a strong decrease compared to the pre-fire image (Figure 8a), with the 2017 summer and winter periods having the lowest values of leaf area index recorded.

**Figure 9:**
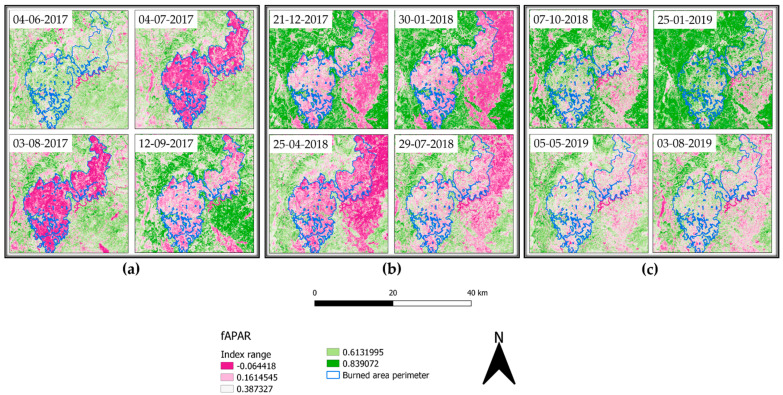
fAPAR representation from 4 June 2017 to 3 August 2019.

**Figure 10:**
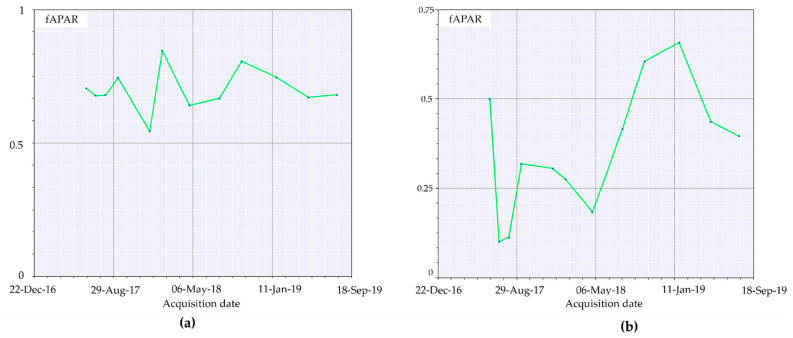
fAPAR index timeseries computed for: (**a**) the burned area (medium severity); (**b**) unburned area.

The trend increased with spring–summer 2018, settling on moderate seasonal fluctuations after January 2019. Using as a reference a nearby unburned area timeseries (Figure 8b), LAI values were almost doubled, showing a natural decrease for wintertime acquisitions and peaks at the end of summer, highlighting the unaffected vegetation behavior. fAPAR is a direct measure of the proportion of incoming photosynthetically active radiation (400–700 nm) absorbed by the canopy. As chlorophyll content is dependent on vegetation health and water, any decrease of it will result in an increase of reflectance. In brief, the index provides a measure of the health of vegetation. Applied to the multitemporal acquisitions of the area of interest (Figure 9a–c), the index performed better compared to LAI for the individuation of unburned patches within the burned area. In the months following the event, it was possible to appreciate the pattern of vegetation activity in the least affected areas. The observation of fAPAR timeseries graph (Figure 10a) showed a small resprouting of bushes and understory, but no leafed vegetation was shown in the LAI timeseries for the second part of summer 2017.

**Figure 11:**
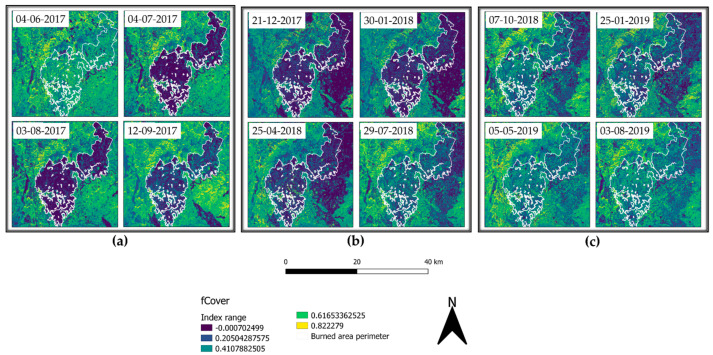
fCover representation from 4 June 2017 to 3 August 2019.

A more evident resprouting of photosynthetically absorbent features as canopies was observed from May 2018 until January 2019 with a successive decrease in summer 2019. Comparing this trend to the unburned area timeseries (Figure 10b), we can appreciate how unaffected vegetation photosynthetically absorbent features reduce over summer to further increase in autumn and winter. fCover corresponds to the gap fraction of vegetation for the nadir direction (Figure 11a–c). Indeed, it is often used to separate vegetation from soil. It is an indicator of plant growth during early periods before the soil is fully canopy covered. From the computed data, it can be observed how in the first year, high severity areas are almost barren.

**Figure 12:**
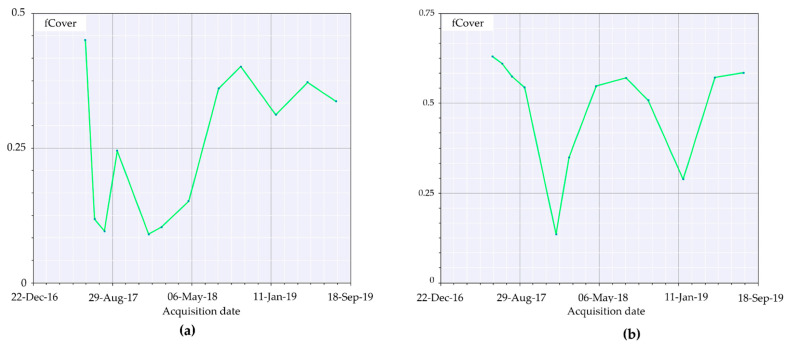
fCover index timeseries computed for: (**a**) the burned area (medium severity); (**b**) unburned area.

The parameter agrees with the fAPAR trend, and healthy vegetation re-establishment providing remote-sensing appreciable ground cover manifested itself with relevance starting from spring 2018. Additionally, seasonal low vegetation elements cyclically appeared at the beginning of spring, reaching the apex in summer and further decreasing in winter and autumn. This tendency aligns with the other computed indices and the phenological phases of vegetation. To better understand the trend reported in the burned area timeseries (Figure 12a) we first had to observe the unburned area tendency (Figure 12b).

The fraction of vegetation covering soil followed the general trend of minimum values for winter and smooth peaks starting from spring to the end of summer, with values around 0.65.

In contrast, the burned area timeseries only showed an increase in fCover after spring 2018 and still presented values 0.20 lower than the same acquisition unburned vegetation.

#### 5.1.3. Hyperspectral Burned Scar Detection Assessment

**Figure 13:**
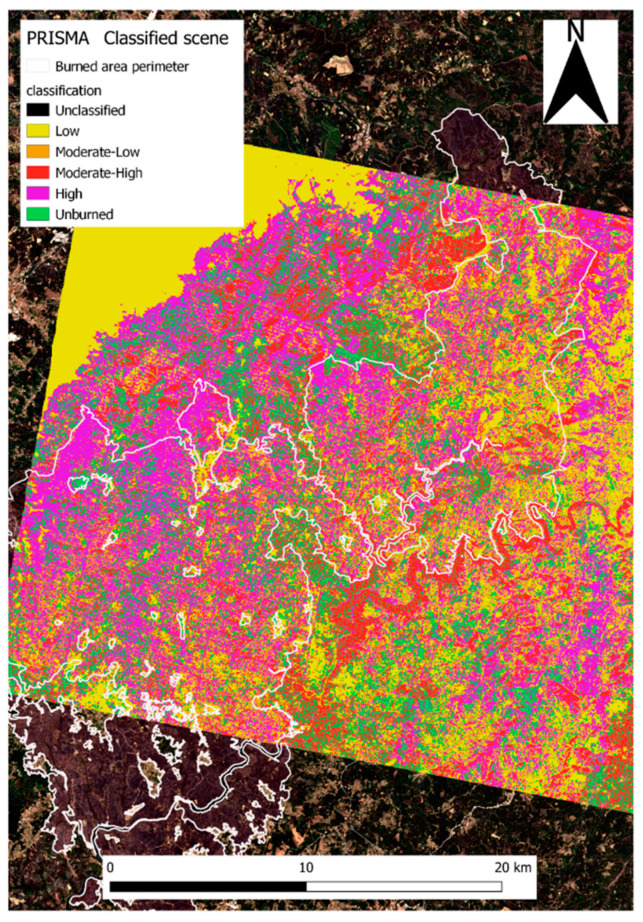
PRISMA image classified based on the RBR severity classes spectral library.

The classified hyperspectral image (Figure 13) provided an adequate individuation of high severity areas, especially in the northwest portion of the fire perimeter. Unburned vegetation patches within the fire perimeter were correctly classified. However, in contrast with this, the area outside the fire perimeter was also classified as previously affected by high severity.

**Figure 14:**
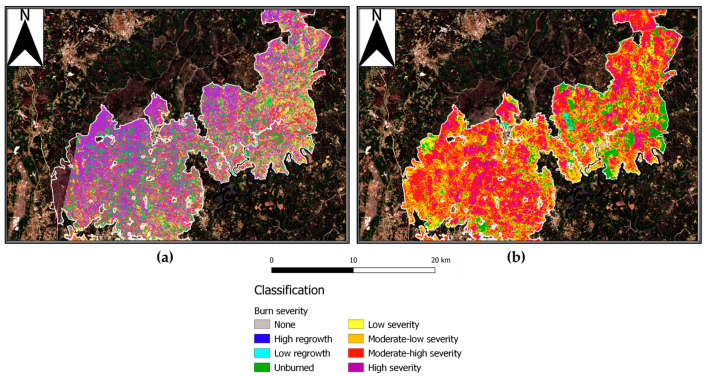
(**a**) PRISMA image classified based on the RBR severity classes spectral library with the application of a fire perimeter mask; (**b**) Sentinel-2 RBR severity map with the fire perimeter mask.

**Figure 15:**
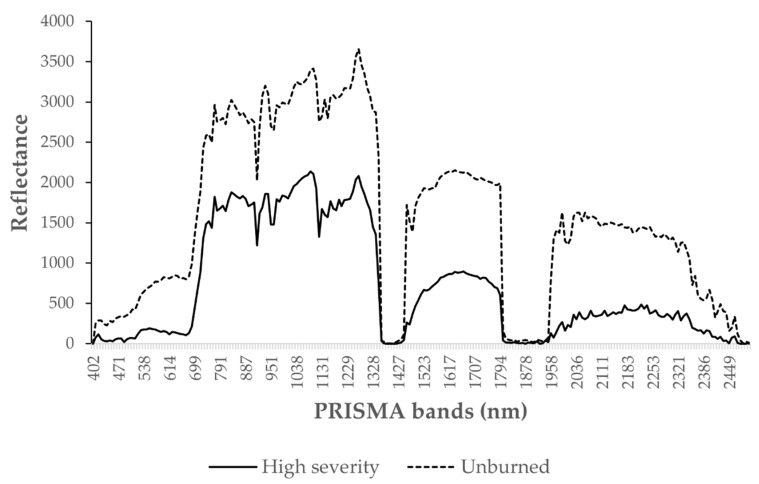
PRISMA scene spectral differences between high-severity areas and unburned parcels. The aim of the image is to represent the difference in extreme scenarios. Spectral signatures vary within the same observed categories.

Given the amount of time elapsed between the fire and the image acquisition, the actual classification of the whole scene was not precise enough to delimitate the fire perimeter nor severity. This meant that in order to achieve a correct classification, Prisma needed to compare the burnt area with a previously computed fire perimeter image. The abovementioned classification could work as a guideline for the determination of the fire effects on natural spectral signatures. Indeed, extreme classes as high burn severity and unburned areas were accurately detected. Nevertheless, the classification of intermediate classes was partially impaired by the vegetation resprouting phenomena. To determine whether the classification performed better within the fire-affected area, we used a mask of the fire perimeter to run the tool. This fire perimeter-bounded classification (Figure 14) performed better regarding the extent of homogeneous classified areas. Indeed, if in the whole scene classification (Figure 13) only extreme classes were correctly classified, with the application of the mask we saw an improvement in the capability of mapping intermediate classes. The results suggest that in order to increase the accuracy of the classification, the use of a mask could improve the accuracy of the classification. This could be due to the fire spectral signature modification combined with vegetation resprouting, occurring from the fire event until the image acquisition. This scenario is a complex spectral mix where charred soil and tree stumps signatures contribute with the newly settled vegetation ones, creating mixed signatures variable in reflectance and depending on the fire severity of the area. As an example, high-severity areas showed lower NIR reflectance compared to healthy vegetation, whereas vegetation showed higher NIR values and lower SWIR ranges (Figure 15) [83].

Overall, high-burn severity areas suffer marked modifications regarding soil denudation and charring resulting in a longer modification of their spectral behavior. In Figure 15, the areas interested by intermediate severity classes were more abundant and better resembled the Sentinel-2 RBR distribution. Generally, the classification workflow proved to find resemblance among the spectra of the different classes. This can be inferred by Figure 15 homogeneity and the statistical validation provided by the tool itself. The accuracy and probability of correct classification is reported, highlighting the accuracy for extreme classes with less sound classification for intermediate values. The lower performance in intermediate classes can be addressed to the variability in the acquired spectra as lower severity resulted in less alteration and vegetation destruction. However, fire severity, post-fire weather conditions, topography, and soil properties were all factors that influenced recovery patterns and therefore the areas’ spectral signatures [84]. As shown above, PRISMA accuracy in assessing the burns scar was not sufficiently performing through the application of the classification workflow tool. Nevertheless, the tool proved how spectral modifications operated by fire persisted through time and affected vegetation spectral signatures growing in the same area.

### 5.2. Study Site 2

#### UAV NDVI Burned Area Mapping

**Figure 16:**
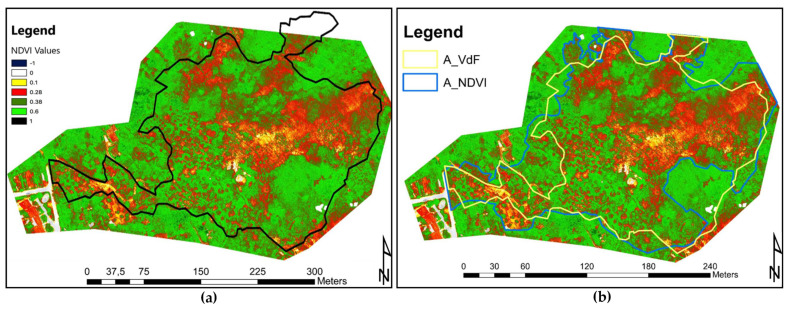
(**a**) Computed NDVI index on the area orthophoto. (**b**) Comparison of the burned area mapped via the field GPS by VdF personnel (yellow polygon), and area mapped by the drone-acquired data (blue polygon).

Future applications of the methodology will be conducted reducing the high resolution used in the present study (2-cm ground resolution) by adopting a greater flight altitude (adapted depending on the morphology of the study area), to favor the number of landmarks in the scene present. This would allow a more efficient image composition for the creation of the orthophoto, reducing the peripheral data absence, and also affecting the reported case study when compared to a portion of the ground mapped fire perimeter. As witnessed by local fire fighters, the fire path was not regular nor linear, since the spreading of the flames was operated by “scattered” sparks. The most affected parcels, located in the center of the burned area, showed evidence of high combustion temperatures as witnessed by the melting of what was supposed to be metal tin. Similarly, in the same parcel and from there uphill, the soil underwent a partial charring, highlighting medium fire severity. From the center of the area, going towards the crest of the hill, the vegetation and soil were heavily affected. Figure 16a, representing the fire-affected vegetation via the NDVI index, reports different and variable index values, reflecting the uneven fire burning path combined with post-fire revegetation and unburned parcels. The first reason for an increased index value could be addressed to the sprouting of ferns, *Robinia*, heather, and generally grass. Secondly, the uneven fire burning path could be a consequence of the firefighter’s effort, aerial water, and flame-retardant throws resulting in an irregular fire-burning path. Even if the regrowth of grass and low bushes did not cover the evidence of fire, they affected the vegetation index, covering the otherwise barren soil and increasing the image noise. From this problem arises the necessity to perform the data sampling as soon as possible after the fire. Figure 16b displays the polygon created by the fire fighters through GPS perimeter mapping, and the polygon deriving from the NDVI index discrimination drawing. Excluding the parcel that was not mapped by the drone flight, the area crafted by the VdF (fire fighters brigade) has a surface extension of 33,328 m^2^, while the one mapped following the false-color representation of the index accounts an area of 36,744 m^2^. Morphologically, the perimeter of the two areas was somehow similar. The greatest difference was the detail level that the NDVI index allows.

**Figure 17:**
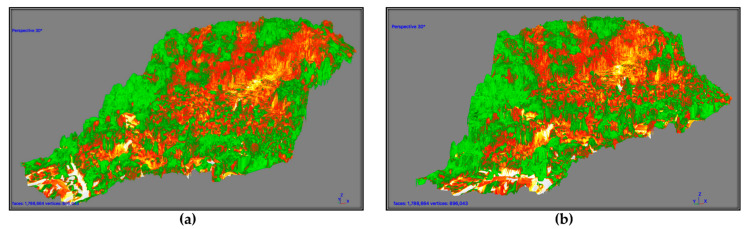
Photoscan 3D mesh of the area with computed NDVI index. (**a**) S view; (**b**) S-W view.

**Figure 18:**
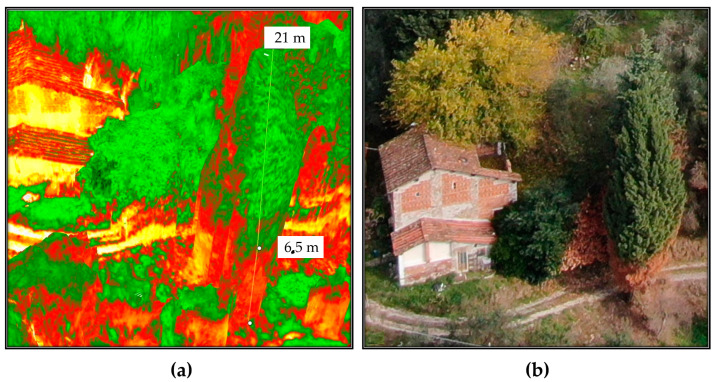
(**a**) NDVI flame height evidence; (**b**) optical flame height.

The processing of the orthophoto resulting in a point cloud creating a 3D representation of the NDVI index (Figure 17a,b) highlighted that trees in different locations of the area have suffered different flame heights. This evidence comes from the neat division of the plant NDVI index. As an example, the 3D visualization of scorched vegetation delineated a clear difference in the index value, the lower portion of a tree that suffered the fire heat had an index amenable to dead vegetation, while the upper part of it had a healthy vegetation index value (Figure 18a).

## 6. Discussion

### 6.1. Study Site 1

The application of remotely sensed data was foreseen simultaneously with a field data validation campaign that could not be performed because of the COVID-19 pandemic. Nevertheless, the available data are still meaningful enough to address and uphold the discussion here proposed. We validated thorough comparison the recovery of the slope cover via Sentinel-2 and applying PRISMA remotely sensed data to evaluate their discrimination power to investigate burned areas. Although Sentinel-2 is widely adopted in research on post-fire related assessment [45], to our knowledge, this is the first study which adopted PRISMA data for acquiring additional hyperspectral information comparing it to Sentinel-2 computed indices. As reported in the results section, Sentinel-2 RBR has provided meaningful information regarding the fire severity that affected the area (Figure 6a,b), while most fire severity indices provide information only after the event RBR proved to be a useful instrument for the monitoring of vegetation recovery after the fire (Figure 6c–f). The applied classification allowed us to investigate the vegetation regrowth within the fire areas, making it possible to observe a phenomenon not previously considered, such as the immediate grass and shrubs resprouting following the fire (Figure 6b). The validation of the observations conduced with RBR has been performed through the comparison of biophysical indices as LAI fAPAR and fCover. All the computed parameters highlighted the temporal extent with which fire affected the area. Despite resprouting, as pointed out by the index graphs, two years after the event vegetation was still suffering lower leaf area index (Figure 7a–c; Figure 8a,b) and lower chlorophyll content (Figure 9a–c; Figure 10a,b). The indices agreed with the RBR temporal trend, and additionally allowed us to observe the phenological stages of vegetation: growth, diffusion, and greater chlorophyll content in spring until midsummer with a quiescence stage in autumn and winter. However, the establishment of stable and arboreal dominant vegetation ground cover was observed only after spring 2018 and still presented values 0.20 lower than unburned vegetation (Figure 11a–c; Figure 12a,b).

The variations in soil properties, vegetation health, and recovery rates induced by fire determined an alteration of the abovementioned features, both in the land cover patterns and in their spectral signatures, as suggested by literature [85]. To assess the ability of PRISMA to detect the effects of fire on the area of interest, we examined the correlation between Sentinel-2 fire severity classes with PRISMA hyperspectral signatures, with pixel-by-pixel comparison. This process led to an empirical classification of hyperspectral signatures that in turn led to a converging classification for burn severity as that computed via Sentinel-2. To our knowledge, this methodology is innovative since it provides an indication on ranges of hyperspectral signatures converging with Sentinel-2 data. The evidence collected with PRISMA proved that despite the elapsed time from the fire event until the acquisition, the alteration of the soil and vegetation persisted, highlighting a different recovery time depending on the severity the area was affected by. Additionally, PRISMA proved to be a valuable instrument for the determination of the residual fire severity in the investigated area basing the observations on the capability of the instrumentation and tool to correctly individuate different severity affected areas. Although this might be true, the application of such procedure must be aided with the adoption of a fire perimeter mask (Figure 15). However, because of the elapsed time from the event to the acquisition, hyperspectral images should be used with caution in order to evaluate burned areas after wildfires. If this information is to be used in postfire decision and on soil stabilization and rehabilitation, then timely data acquisitions and analysis are essential [85].

### 6.2. Study Site 2

UAV burned area analysis can hardly take advantage of multitemporal acquisitions for the computation of severity indices as its application is site-specific and “on demand”, usually adopted after the event [86]. Nevertheless, this complexity is overcome with the increased detail and completeness of information deriving from the high-resolution sensors [87]. Consequently, indices computed from these images allowed for a more precise distinction of the thresholds necessary for the separation of different severity classes in the study area. This achievement, combined with ANNs (artificial neural network), proved to be an effective solution for burned area mapping and active fire individuation, also when tested with field validation [88]. Oftentimes, the adopted UAVs are military-grade vehicles equipped with multispectral sensors, specifically modified for the task [89]. The range of sensors these platforms can fit depends on the carrying capacity and the effective benefit they can provide to the analysis. Indeed, multispectral sensors can be coupled with LiDAR, thermal, optical, and hyperspectral sensors [90,91,92,93].

Burned area mapping is operated to determine the extent of the burned area and to investigate fire severity affecting the soil–vegetation interface. Different fire severity classes correspond to heterogeneous modifications of soil and vegetation. The effects of fire severity on the soil and vegetal fractions cause the removal of vegetative cover, soil water repellency, and increase soil erodibility as a consequence of organic matter and root breakdown. All of these factors combined strongly influence soil hydrological budget components as runoff, infiltration, and evapotranspiration [94,95]. These parameters influencing the response of burned areas over time result in a modification of hydrogeological phenomena processes. These phenomena are landslides, debris flows, and general slope instability events [96]. Therefore, UAV multi-sensor acquisitions are strongly beneficial in the assessment of the parameters influencing slope instability processes.

The adopted UAV mapping procedure proved to be a powerful tool in the burned area mapping due to its quickness of deployment, data collection, and accuracy. The three-dimensional representation of the orthophoto via a dense point cloud allowed us to investigate the effects of fire on different vertical sections of the vegetative cover, highlighting the influence of hill aspect, slope, and vegetation structure, Figure 16 and Figure 17 [97]. The possibility to visualize the NDVI index value on the point cloud allowed the selection and removal of points not representing the investigated area. As an example, to map the area using as discriminant the NDVI value of the ground cover, we could filter out the points that were over a certain z quota to obtain an index only representing the understory. Nevertheless, this result was obtained with image acquisition geometry performed with the sensor tilted towards the vertical elements of the woods, acquiring an under-the-canopy perspective. Generally, as the vegetation cover is dynamic and fast changing and experienced with PRISMA, timely acquisitions strongly influence the performance of the procedure. Indeed, the immediate application of the method after the fire would have resulted in less vegetation resprouting (*Robinia*, fern), reducing the index blurring and favouring a neater severity analysis and index distinction.

The burned area perimeter delimitation computed via NDVI and ground-based mapping resulted in products differing for shape, detail, boundary position, and areal extent. The main differences in the boundary position were recorded in portions of the area with harsh morphology. In these areas, fire fighters’ ground-based mapping included within the burned perimeter unaffected portions, as they adopted a compromise between burn marks and area accessibility. This difference can be only acknowledged by the comparison with UAV-based NDVI. In addition, the higher detail of the remotely sensed perimeter allowed for a more precise positioning of the burned area boundary. This aspect, combined with the possibility to separate crown and ground NDVI values in UAV mapping, could address the main differences between the two areas.

A future advance of the procedure will comprehend a hyperspectral UAV-mounted sensor, acquiring high-resolution spectral images of the scene, useful for further chemically based analysis and classification of the scene, resulting in a more precise mapping.

### 6.3. Comparison of Satellite and UAV Results and Future Developments

Both case studies’ burn severity was analyzed via the computation of spectral indices, RBR and NDVI. Despite the two indices having different mathematical structure and temporal parameters, they both analyzed land cover status after wildfires. Satellite-based data has shown the advantages of modern sensing platforms, short revisit time, great areal coverage, and medium–high resolution, allowing for the investigation of extensive phenomena as wildfires and ecosystem recovery. The RBR analysis highlighted a stark contrast in the severity classes coexisting in a small area. The presence of clashing classes confining with each other could be addressed to the spatial extent of pixels. In contrast, UAV-computed NDVI data efficiently represented the transition, even shortly extended, occurring among different severity classes. The ability to represent this phenomenon could be addressed to the ultra-high UAV data resolution. This discrepancy could be addressed to scale factor, where, depending on the observation scale, processes which appear homogeneous at a local scale may become heterogeneous at a large scale [98,99,100]. The scale effect is widely known to be a problematic phenomenon in the remote sensing of environment and needs specific calibration to reduce its effect on the analysis [101,102,103]. As land surface is very heterogeneous, upscaling data validation (from large scale to local scale) is more relevant than down-scaling (from local scale to large scale) because of the generalization performed to scale data up [104]. In this optic, the proposed methodology theoretically analyzed the possible benefit of drone acquired data for satellite product detail enhancement. Indeed, the UAV acquisition pointed out its versatility in acquiring almost ground level data. This capability, combined with the modularity of the drone sensor bay to host multiple sensors, opens new possibilities to adopt the UAV platform as a satellite validation tool. Furthermore, as multispectral and hyperspectral satellites provide limited ground spatial resolution and suffer from atmospheric interactions phenomena, if correctly scaled, the synergy with UAV-sensed data could create a measurement-validation multiplatform procedure.

## 7. Conclusions

The overall proposed procedure proved to be a valuable and efficient tool to determine post-fire vegetation recovery and wildfire-burned area analysis. The applied Sentinel-2 RBR index severity classification demonstrated the efficiency of the index in mapping diverse severity classes and burn patterns without reducing the performance for unburned parcels and peripheral areas. Sentinel-2 RBR index multitemporal analysis highlighted the different vegetation response as a function of the fire severity that affected the area, recording both the immediate shrub resprouting following the fire, the phenological resting phase of the vegetation and its development in the years following the fire. The RBR results tested with the biophysical computed parameters showed concordance regarding the vegetation indices trend. The two year-long analyses allowed us to observe how high severity areas underwent a rapid sprouting phenomenon following the fire, contrasting with the later acquisitions where the same areas suffered from lower fCover and LAI if compared to intermediate severity classes. The hyperspectral satellite adopted embedded challenges in its data-processing arising from the novelty of the platform and data complexity. Nevertheless, despite the proposed method being an exploratory approach, the analysis highlighted the satellite performance and high spectral resolution. PRISMA ability to detect spectral signature changes in fire-affected areas proved to be efficient regarding high severity areas despite the elapsed time, testifying the alteration of soil and vegetation persistence, highlighting a different recovery time depending on the severity the area was affected by. The innovative multiplatform methodology proposed was performed with the correlation between Sentinel-2 RBR fire severity classes and the scene hyperspectral signature, performed with a pixel-by-pixel comparison, leading to a converging classification. The UAV NDVI index provided high-resolution data that could be integrated with the proposed methodology in study site two to benefit the overall accuracy and completeness of information. All the adopted platforms benefitted from a timely data acquisition for a representative data analysis. Future developments will focus on the integration between drone hyperspectral images and satellite hyperspectral data to create a multiplatform and multiscale scene analysis, increasing the overall accuracy. Indeed, the drone acquired data will provide a hyperspectral “ground-like” information useful for hyperspectral satellite acquired data scene classification.

## Figures and Tables

**Table 1:** Sentinel-2 MSI sensing bands range and purpose (Rn., range; Res., resolution; WL, wavelength) [20].

Nr.	Name	Start WL	Middle WL	End WL	Spectral Rn.	Spatial Res.	Purpose
1	1	433	443	453	20	60	Atmospheric correction (aerosol scattering)
2	2	458	490	522	65	10	Sensitive to vegetation senescing, carotenoid, browning, and soil background; atmospheric correction (aerosol scattering)
3	3	543	560	577	35	10	Green peak; sensitive to total chlorophyll in vegetation
4	4	650	665	680	30	10	Maximum chlorophyll absorption
5	5	698	705	712	15	20	Position of red edge; consolidation of atmospheric corrections–fluorescence baseline
6	6	733	740	747	15	20	Position of red edge, atmospheric correction; retrieval of aerosol load
7	7	773	783	793	20	20	LAI, edge of the NIR plateau
8	8	785	842	899	115	10	LAI
9	8a	855	865	875	20	20	NIR plateau; sensitive to total chlorophyll, biomass, LAI, and protein; water vapor absorption reference; retrieval of aerosol load and type
10	9	935	945	955	20	60	Water vapor absorption; atmospheric correction
11	10	1360	1375	1390	30	60	Detection of thin cirrus for atmospheric correction
12	11	1565	1610	1655	90	20	Sensitive to lignin, starch, and forest aboveground biomass; snow–ice–cloud separation
13	12	2100	2190	2280	180	20	Assessment of Mediterranean vegetation conditions; distinction of clay soils for the monitoring of soil erosion; distinction between live biomass, dead biomass, and soil (e.g., for burn scars mapping)

**Table 2:** Prisma hyperspectral and panchromatic sensors specifications. Modified after [49].

PRISMA Sensors Characteristics	
Swath/FOV	30 km/2.45°
GSD	Hyperspectral 30 m
	Panchromatic 5 m
Spatial pixels	Hyperspectral: 1000
	Panchromatic: 6000
Spectral range	VNIR: 400–1010 nm
	SWIR: 920–2505
Spectral resolution	≤12 nm
Spectral bands	VNIR: 66
	SWIR: 171
Radiometric quantization	12 bit
Absolute radiometric accuracy	Better than 5%

**Table 3:** Sentinel-2 images acquisition date and couplets (* pre-fire acquisition).

Acquisition Date	Couplet
04/06/2017 *	J_J_17
04/07/2017	
03/08/2017	A_S_17
12/09/2017	
21/12/2017	D_J_18
30/01/2018	
25/04/2018	A_J_18
29/07/2018	
07/10/2018	O_J_19
25/01/2019	
05/05/2019	M_A_19
03/08/2019	

**Table 4:** RBR range for classification as suggested by USGS. Adapted from [80].

Severity Level	dNBR Range (Not Scaled)
Enhanced regrowth, high (post-fire)	−0.500 to −0.251
Enhanced regrowth, low (post-fire)	−0.250 to −0.101
Unburned	−0.100 to +0.99
Low severity	+0.100 to +0.269
Moderate–low severity	+0.270 to +0.439
Moderate–high severity	+0.440 to +0.659
High severity	+0.660 to +1.300

## Data Availability

All data are available on Copernicus open access hub and PRISMA mission data download portal.

## References

[1] Carrión J.S., Yll E., Walker M.J., Legaz A.J., Chaín C., López A. (2003). Glacial refugia of temperate, Mediterranean and Ibero-North African flora in south-eastern Spain: New evidence from cave pollen at two Neanderthal man sites. Glob. Ecol. Biogeogr..

[2] Walker M.J., Anesin D., Angelucci D.E., Avilés-Fernández A., Berna F., Buitrago-López A.T., Fernández-Jalvo Y., Haber-Uriarte M., López-Jiménez A., López-Martínez M. (2016). Combustion at the late Early Pleistocene site of Cueva Negra del Estrecho del Río Quípar (Murcia, Spain). Antiquity.

[3] Shakesby R.A. (2011). Post-wildfire soil erosion in the Mediterranean: Review and future research directions. Earth-Sci. Rev..

[4] Department of Biodiversity Conservation, Government of Western Australia Fuel Loads and Fire Intensity. Https://www.google.com/search?q=Department+of+Biodiversity+Conservation%2C+Government+of+Western+Australia%2C+%282019%29.+Fuel+loads+and+fire+intensity.&sxsrf=ALeKk01G9ZWIsBvgSGNZjL8DxAk9J6FDVw%3A1621088233415&ei=6defYL_hGIKcsAeqyY6QCQ&oq=Department+of+Bi.

[5] The DRIVER+ Project and CMINE (2020). CMINE Task Group Wildfire Management Final Report.

[6] Wotton B.M., Nock C.A., Flannigan M.D. (2010). Forest fire occurrence and climate change in Canada. Int. J. Wildl. Fire.

[7] Lazzeri G. (2018). Explorative Use of Drone (UAV) Remotely Sensed Data for Quick Mapping of Wildfire Burnt Areas. Bachelor’s Thesis.

[8] Chuvieco E., Riaño D., Danson F.M., Martin P. (2006). Use of a radiative transfer model to simulate the postfire spectral response to burn severity. J. Geophys. Res. Biogeosci..

[9] Pereira J.M.C., Sá A.C.L., Sousa A.M.O., Silva J.M.N., Santos T.N., Carreiras J.M.B. (1999). Spectral characterisation and discrimination of burnt areas. Remote Sensing of Large Wildfires.

[10] Shakesby R.A., Doerr S.H. (2006). Wildfire as a hydrological and geomorphological agent. Earth-Sci. Rev..

[11] Beatty S.M., Smith J.E. (2013). Dynamic soil water repellency and infiltration in post-wildfire soils. Geoderma.

[12] Jensen J.R., Brigham Young University (2007). Remote Sensing of the Environment: An Earth Resource Perspective.

[13] Epting J., Verbyla D., Sorbel B. (2005). Evaluation of remotely sensed indices for assessing burn severity in interior Alaska using Landsat TM and ETM+. Remote Sens. Environ..

[14] Chuvieco E., Mouillot F., van der Werf G.R., San Miguel J., Tanasse M., Koutsias N., García M., Yebra M., Padilla M., Gitas I. (2019). Historical background and current developments for mapping burned area from satellite Earth observation. Remote Sens. Environ..

[15] Seiler W., Crutzen P.J. (1980). Estimates of gross and net fluxes of carbon between the biosphere and the atmosphere from biomass burning. Clim. Chang..

[16] Reilly J., Prinn R., Harnisch J., Fitzmaurice J., Jacoby H., Kicklighter D., Melillo J., Stone P., Sokolov A., Wang C. (1999). Multi-gas assessment of the Kyoto Protocol. Nature.

[17] Pearson L., Pelling M. (2015). The UN Sendai Framework for Disaster Risk Reduction 2015–2030: Negotiation Process and Prospects for Science and Practice. J. Extrem. Events.

[18] Protezione Civile Description of the Fire Risk. http://www.protezionecivile.gov.it/jcms/it/descrizione_incendi.

[19] Aschbacher J., Milagro-Pérez M.P. (2012). The European Earth monitoring (GMES) programme: Status and perspectives. Remote Sens. Environ..

[20] ESA (2013). Sentinel-2 User Handbook.

[21] European Space Agency (ESA) (2017). Sentinel-2 Spectral Response Functions (S2-SRF).

[22] Filipponi F. (2018). BAIS2: Burned Area Index for Sentinel-2. Proceedings.

[23] Stavrakoudis D.G., Katagis T., Minakou C., Gitas I.Z. Towards a fully automatic processing chain for operationally mapping burned areas countrywide exploiting Sentinel-2 imagery. Proceedings of the Seventh International Conference on Remote Sensing and Geoinformation of the Environment (RSCy2019).

[24] Pepe M., Parente C. (2018). Burned area recognition by change detection analysis using images derived from Senti-nel-2 satellite: The case study of Sor-rento Peninsula, Italy. J. Appl. Eng. Sci..

[25] Roteta E., Bastarrika A., Padilla M., Storm T., Chuvieco E. (2019). Development of a Sentinel-2 burned area algorithm: Generation of a small fire database for sub-Saharan Africa. Remote Sens. Environ..

[26] Roy D.P., Huang H., Boschetti L., Giglio L., Yan L., Zhang H.H., Li Z. (2019). Landsat-8 and Sentinel-2 burned area mapping—A combined sensor multi-temporal change detection approach. Remote Sens. Environ..

[27] Quintano C., Fernández-Manso A., Fernández-Manso O. (2018). Combination of Landsat and Sentinel-2 MSI data for initial assessing of burn severity. Int. J. Appl. Earth Obs. Geoinf..

[28] Loizzo R., Guarini R., Longo F., Scopa T., Formaro R., Facchinetti C., Varacalli G. Prisma: The Italian hyperspectral mission. Proceedings of the International Geoscience and Remote Sensing Symposium (IGARSS).

[29] Smith D. (2021). Abstracts of the 44th Mineral Deposits Study Group Annual Winter Meeting held virtually on 14th December 2020. Appl. Earth Sci..

[30] Vangi E., D’amico G., Francini S., Giannetti F., Lasserre B., Marchetti M., Chirici G. (2021). The new hyperspectral satellite prisma: Imagery for forest types discrimination. Sensors.

[31] Cusworth D.H., Duren R.M., Thorpe A.K., Pandey S., Maasakkers J.D., Aben I., Jervis D., Varon D.J., Jacob D.J., Randles C.A. (2021). Multisatellite Imaging of a Gas Well Blowout Enables Quantification of Total Methane Emissions. Geophys. Res. Lett..

[32] Sharpe J.R. (1931). The Forest Resources of Ontario, 1930.

[33] Kolden C.A., Weisberg P.J. (2007). Assessing Accuracy of Manually-mapped Wildfire Perimeters in Topographically Dissected Areas. Fire Ecol..

[34] Hitchcock H.C., Hoffer R.M. Mapping a recent forest fire with ERTS-1 MSS data. Proceedings of the Remote Sensing of Earth Resources: Volume 3—Third Conference on Earth Resources Observation and Information Analysis System.

[35] Filipponi F. (2019). Exploitation of sentinel-2 time series to map burned areas at the national level: A case study on the 2017 Italy wildfires. Remote Sens..

[36] Filipponi F., Manfron G. (2019). Observing Post-Fire Vegetation Regeneration Dynamics Exploiting High-Resolution Sentinel-2 Data. Proceedings.

[37] Sobrino J.A., Llorens R., Fernández C., Fernández-Alonso J.M., Vega J.A. (2019). Relationship between forest fires severity measured in situ and through remotely sensed spectral indices. Forests.

[38] García-Llamas P., Suárez-Seoane S., Fernández-Guisuraga J.M., Fernández-García V., Fernández-Manso A., Quintano C., Taboada A., Marcos E., Calvo L. (2019). Evaluation and comparison of Landsat 8, Sentinel-2 and Deimos-1 remote sensing indices for assessing burn severity in Mediterranean fire-prone ecosystems. Int. J. Appl. Earth Obs. Geoinf..

[39] Bonis R.D., Laneve G. Development of a Vegetation Damage Severity Index for the Italian Hyperspectral Sensor PRISMA. https://www.semanticscholar.org/paper/Development-of-a-vegetation-damage-severity-index-Bonis-Laneve/7590a757055c279a2bc07896da2fa522dadce414.

[40] Chen D., Loboda T.V., Hall J.V. (2020). A systematic evaluation of influence of image selection process on remote sensing-based burn severity indices in North American boreal forest and tundra ecosystems. ISPRS J. Photogramm. Remote Sens..

[41] Suresh Babu K.V., Roy A., Aggarwal R. (2018). Mapping of forest fire burned severity using the Sentinel datasets. ISPRS Int. Arch. Photogramm. Remote Sens. Spat. Inf. Sci..

[42] Cocke A.E., Fulé P.Z., Crouse J.E. (2005). Comparison of burn severity assessments using Differenced Normalized Burn Ratio and ground data. Int. J. Wildl. Fire.

[43] Samiappan S., Hathcock L., Turnage G., McCraine C., Pitchford J., Moorhead R. (2019). Remote sensing of wildfire using a small unmanned aerial system: Post-fire mapping, vegetation recovery and damage analysis in grand bay, Mississippi/Alabama, USA. Drones.

[44] Fernández-Guisuraga J.M., Sanz-Ablanedo E., Suárez-Seoane S., Calvo L. (2018). Using unmanned aerial vehicles in postfire vegetation survey campaigns through large and heterogeneous areas: Opportunities and challenges. Sensors.

[45] Fernández-Manso A., Fernández-Manso O., Quintano C. (2016). SENTINEL-2A red-edge spectral indices suitability for discriminating burn severity. Int. J. Appl. Earth Obs. Geoinf..

[46] Curran P.J., Dungan J.L., Gholz H.L. (1990). Exploring the relationship between reflectance red edge and chlorophyll content in slash pine. Tree Physiol..

[47] Demattê J.A.M., da Silva Terra F. (2014). Spectral pedology: A new perspective on evaluation of soils along pedogenetic alterations. Geoderma.

[48] ASI Hyperspectral Satellite, Capable of Observing from the Optical to the Near Infrared. https://www.asi.it/en/earth-science/prisma/.

[49] Barducci A., Di Ninni P., Guzzi D., Lastri C., Nardino V., Pippi I., Raimondi V. The OPTIMA project: Data simulation and correction procedures for PRISMA mission products. Proceedings of the Sensors, Systems, and Next-Generation Satellites XVIII.

[50] Vermeulen C., Lejeune P., Lisein J., Sawadogo P., Bouché P. (2013). Unmanned Aerial Survey of Elephants. PLoS ONE.

[51] Puliti S., Ørka H.O., Gobakken T., Næsset E. (2015). Inventory of small forest areas using an unmanned aerial system. Remote Sens..

[52] Rossi G., Nocentini M., Lombardi L., Vannocci P., Tanteri L., Dotta G., Bicocchi G., Scaduto G., Salvatici T., Tofani V. (2019). Integration of multicopter drone measurements and ground-based data for landslide monitoring. Landslides and Engineered Slopes. Experience, Theory and Practice.

[53] Gupta S.K., Shukla D.P. (2018). Application of drone for landslide mapping, dimension estimation and its 3D reconstruction. J. Indian Soc. Remote Sens..

[54] Rossi G., Tanteri L., Tofani V., Vannocci P., Moretti S., Casagli N. (2017). Brief Communication: Use of multicopter drone optical images for landslide mapping and characterization. Nat. Hazards Earth Syst. Sci. Discuss.

[55] Drones Imaging NDVI Cameras. https://www.dronesimaging.com/en/solutions/ndvi-cameras/.

[56] Meddens A.J.H., Kolden C.A., Lutz J.A. (2016). Detecting unburned areas within wildfire perimeters using Landsat and ancillary data across the northwestern United States. Remote Sens. Environ..

[57] Rossi G., Tanteri L., Tofani V., Vannocci P., Moretti S., Casagli N. (2018). Multitemporal UAV surveys for landslide mapping and characterization. Landslides.

[58] Dawson T.P., North P.R.J., Plummer S.E., Curran P.J. (2003). Forest ecosystem chlorophyll content: Implications for remotely sensed estimates of net primary productivity. Int. J. Remote Sens..

[59] García M.J.L., Caselles V. (1991). Mapping burns and natural reforestation using thematic mapper data. Geocarto Int..

[60] Basso B., Cammarano D., De Vita P. (2004). Remotely sensed vegetation indices: Theory and applications for crop management. Riv. Ital. Agrometeorol..

[61] Parks S.A., Dillon G.K., Miller C. (2014). A new metric for quantifying burn severity: The relativized burn ratio. Remote Sens..

[62] Miller J.D., Thode A.E. (2007). Quantifying burn severity in a heterogeneous landscape with a relative version of the delta Normalized Burn Ratio (dNBR). Remote Sens. Environ..

[63] Serco Italia SPA Burned Area Mapping with Sentinel-2 (SNAP), Portugal (Version 1.2). Retrieved from RUS Lectures. https://rus-copernicus.eu/portal/the-rus-library/learn-by-yourself/.

[64] Pan H., Chen Z., Ren J., Li H., Wu S. (2019). Modeling Winter Wheat Leaf Area Index and Canopy Water Content with Three Different Approaches Using Sentinel-2 Multispectral Instrument Data. IEEE J. Sel. Top. Appl. Earth Obs. Remote Sens..

[65] Zuhlke M., Fomferra N., Brockmann C., Peters M., Veci L., Malik J., Regner P. SNAP (Sentinel Application Platform) and the ESA Sentinel 3 Toolbox—NASA/ADS. Proceedings of the Sentinel-3 for Science Workshop.

[66] QGIS.org QGIS Geographic Information System. https://guides.nyu.edu/gis/qgis.

[67] Weiss M., Baret F. (2016). S2ToolBox Level 2 Products: LAI, FAPAR, FCOVER.

[68] DGADR Direção-Geral de Agricultura e Desenvolvimento Rural. https://www.dgadr.gov.pt/index.php?option=com_content&view=article&id=342.

[69] Climate-Data.org Castanheira de Pera Climate. https://en.climate-data.org/europe/portugal/castanheira-de-pera/castanheira-de-pera-882485/.

[70] Vega J.A., Fontúrbel T., Merino A., Fernández C., Ferreiro A., Jiménez E. (2013). Testing the ability of visual indicators of soil burn severity to reflect changes in soil chemical and microbial properties in pine forests and shrubland. Plant Soil.

[71] Regione Toscana Incendio Boschivo a Vinchiana, Lucca—Toscana Notizie. https://www.toscana-notizie.it/-/incendio-boschivo-a-vinchiana-lucca.

[72] Climate-Data.org Clima Lucca: Temperatura, Medie Climatiche, Pioggia Lucca. Grafico Pioggia e Grafico Temperatura Lucca—Climate-Data.org. https://it.climate-data.org/europa/italia/tuscany/lucca-718595/.

[73] EU (2016). Copernicus programme European Digital Elevation Model (EU-DEM).

[74] Sentinel Hub Copernicus Sentinel-2 Modified Data, Processed with EO. https://apps.sentinel-hub.com/eo-browser/?zoom=11&lat=39.97107&lng=-8.22395&themeId=WILDFIRES-NORMAL-MODE&visualizationUrl=https%3A%2F%2Fservices.sentinel-hub.com%2Fogc%2Fwms%2Faae18701-6b25-4001-8b2a-b98a1b3806c1&datasetId=S2L2A&fromTime=2017-07-29T00%3A00%3A00.000Z&toTime=2017-07-29T23%3A59%3A59.999Z&layerId=BURNED-AREAS-DETECTION.

[75] EU (2020). Copernicus Programme Corine Land Cover (CLC) 2012.

[76] EU Copernicus Programme Corine Land Cover (CLC) Classes. https://land.copernicus.eu/Corinelandcoverclasses.eps.75dpi.png/image_view_fullscreen.

[77] Regione Toscana (2019). Uso e Copertura del Suolo, UCS10k 2019—Total Rendering. http://www502.regione.toscana.it/geoscopio/servizi/wms/USO_E_COPERTURA_DEL_SUOLO.htm.

[78] van der Linden S., Rabe A., Held M., Jakimow B., Leitão P.J., Okujeni A., Schwieder M., Suess S., Hostert P. (2015). The EnMAP-box-A toolbox and application programming interface for EnMAP data processing. Remote Sens..

[79] McFeeters S.K. (1996). The use of the Normalized Difference Water Index (NDWI) in the delineation of open water features. Int. J. Remote Sens..

[80] UN-SPIDER Knowledge Portal Normalized Burn Ratio (NBR). https://un-spider.org/advisory-support/recommended-practices/recommended-practice-burn-severity/in-detail/normalized-burn-ratio.

[81] Kokaly R.F., Rockwell B.W., Haire S.L., King T.V.V. (2007). Characterization of post-fire surface cover, soils, and burn severity at the Cerro Grande Fire, New Mexico, using hyperspectral and multispectral remote sensing. Remote Sens. Environ..

[82] Agisoft (2018). PhotoScan—Photogrammetric Processing of Digital Images and 3D Spatial Data Generation. https://www.agisoft.com/.

[83] Veraverbeke S., Dennison P., Gitas I., Hulley G., Kalashnikova O., Katagis T., Kuai L., Meng R., Roberts D., Stavros N. (2018). Hyperspectral remote sensing of fire: State-of-the-art and future perspectives. Remote Sens. Environ..

[84] Pausas J.G., Fernández-Muñoz S. (2012). Fire regime changes in the Western Mediterranean Basin: From fuel-limited to drought-driven fire regime. Clim. Chang..

[85] Robichaud P.R., Lewis S.A., Laes D.Y.M., Hudak A.T., Kokaly R.F., Zamudio J.A. (2007). Postfire soil burn severity mapping with hyperspectral image unmixing. Remote Sens. Environ..

[86] Whitehead K., Hugenholtz C.H. (2014). Remote sensing of the environment with small unmanned aircraft systems (Uass), part 1: A review of progress and challenges. J. Unmanned Veh. Syst..

[87] Fraser R.H., van der Sluijs J., Hall R.J. (2017). Calibrating satellite-based indices of burn severity from UAV-derived metrics of a burned boreal forest in NWT, Canada. Remote Sens..

[88] Shin J.I., Seo W.W., Kim T., Park J., Woo C.S. (2019). Using UAV multispectral images for classification of forest burn severity—A case study of the 2019 Gangneung forest fire. Forests.

[89] Ambrosia V.G., Wegener S., Zajkowski T., Sullivan D.V., Buechel S., Enomoto F., Lobitz B., Johan S., Brass J., Hinkley E. (2011). The Ikhana unmanned airborne system (UAS) western states fire imaging missions: From concept to reality (2006–2010). Geocarto Int..

[90] Sankey T., Donager J., McVay J., Sankey J.B. (2017). UAV lidar and hyperspectral fusion for forest monitoring in the southwestern USA. Remote Sens. Environ..

[91] Espinoza C.Z., Khot L.R., Sankaran S., Jacoby P.W. (2017). High resolution multispectral and thermal remote sensing-based water stress assessment in subsurface irrigated grapevines. Remote Sens..

[92] Frodella W., Elashvili M., Spizzichino D., Gigli G., Adikashvili L., Vacheishvili N., Kirkitadze G., Nadaraia A., Margottini C., Casagli N. (2020). Combining infrared thermography and UAV digital photogrammetry for the protection and conservation of rupestrian cultural heritage sites in Georgia: A methodological application. Remote Sens..

[93] Frodella W., Lazzeri G., Moretti S., Keizer J., Verheijen F.G.A. (2020). Applying infrared thermography to soil surface temperature monitoring: Case study of a high-resolution 48 h survey in a vineyard (Anadia, Portugal). Sensors.

[94] Lourenco L., Nunes A., Bento-Goncalves A., Vieir A. (2012). Soil Erosion After Wildfires in Portugal: What Happens When Heavy Rainfall Events Occur?. Research on Soil Erosion.

[95] Araújo Santos L.M., Correia A.J.P.M., Coelho P.A.L.F. (2020). Post-wildfire slope stability effects and mitigation: A case study from hilly terrains with unmanaged forest. SN Appl. Sci..

[96] Ozturk U., Tarakegn Y.A., Longoni L., Brambilla D., Papini M., Jensen J. (2016). A simplified early-warning system for imminent landslide prediction based on failure index fragility curves developed through numerical analysis. Geomat. Nat. Hazards Risk.

[97] Agee J.K. (1993). Fire Ecology of Pacific Northwest Forests.

[98] Cao C.Y., Lam N., Quattrocchi D.A., Goodchild M.F. (1997). Understanding the scale and resolution effects in remote sensing and GIS. Scale in Remote Sensing and GIS.

[99] Bian L., Quattrocchi D.A., Goodchild M.F. (1997). Multiscale nature of spatial data in scaling up environmental models. Scale in Remote Sensing and GIS.

[100] Finke P.A., Bierkens M.F., De Willigen P. (2002). Upscaling and Downscaling Methods for Environmental Research.

[101] Hufkens K., Bogaert J., Dong Q.H., Lu L., Huang C.L., Ma M.G., Che T., Li X., Veroustraete F., Ceulemans R. (2008). Impacts and uncertainties of upscaling of remote-sensing data validation for a semi-arid woodland. J. Arid Environ..

[102] Su L., Li X., Liang S., Strahler A.H. (2003). Simulation of scaling effects of thermal emission from non-isothermal pixels with the typical three-dimensional structure. Int. J. Remote Sens..

[103] Jiang Z., Huete A.R., Chen J., Chen Y., Li J., Yan G., Zhang X. (2006). Analysis of NDVI and scaled difference vegetation index retrievals of vegetation fraction. Remote Sens. Environ..

[104] Wu H., Li Z.L. (2009). Scale issues in remote sensing: A review on analysis, processing and modeling. Sensors.

